# Provider initiated tuberculosis case finding in outpatient departments of health care facilities in Ghana: yield by screening strategy and target group

**DOI:** 10.1186/s12879-017-2843-5

**Published:** 2017-12-01

**Authors:** Sally-Ann Ohene, Frank Bonsu, Nii Nortey Hanson-Nortey, Ardon Toonstra, Adelaide Sackey, Knut Lonnroth, Mukund Uplekar, Samuel Danso, George Mensah, Felix Afutu, Paul Klatser, Mirjam Bakker

**Affiliations:** 1 World Health Organization Country Office, 29 Volta Street Airport, Airport Residential Area, P.O. Box MB 142 Accra, Ghana; 2 National Tuberculosis Control Program, Accra, Ghana; 3 KIT Health, Royal Tropical Institute (KIT), Amsterdam, The Netherlands; 4 Global TB Programme, WHO, Geneva, Switzerland; 5 Ghana Health Service, Accra, Ghana; 6 Department of Global Health, Academic Medical Centre, Amsterdam Institute of Global Health and Development, Amsterdam, The Netherlands

**Keywords:** Tuberculosis, Screening, Case finding, Ghana

## Abstract

**Background:**

Meticulous identification and investigation of patients presenting with tuberculosis (TB) suggestive symptoms rarely happen in crowded outpatient departments (OPDs). Making health providers in OPDs diligently follow screening procedures may help increase TB case detection. From July 2010 to December 2013, two symptom based TB screening approaches of varying cough duration were used to screen and test for TB among general outpatients, PLHIV, diabetics and contacts in Accra, Ghana.

**Methods:**

This study was a retrospective analysis comparing the yield of TB cases using two different screening approaches, allocated to selected public health facilities. In the first approach, the conventional 2 weeks cough duration with or without other TB suggestive symptoms was the criterion to test for TB in attendants of 7 general OPDs. In the second approach the screening criteria cough of >24 hours, as well as a history of at least one of the following symptoms: fever, weight loss and drenching night sweats were used to screen and test for TB among attendants of 3 general OPDs, 7 HIV clinics and 2 diabetes clinics. Contact investigation was initiated for index TB patients. The facilities documented the number of patients verbally screened, with presumptive TB, tested using smear microscopy and those diagnosed with TB in order to calculate the yield and number needed to screen (NNS) to find one TB case. Case notification trends in Accra were compared to those of a control area.

**Results:**

In the approach using >24-hour cough, significantly more presumptive TB cases were identified among outpatients (0.82% versus 0.63%), more were tested (90.1% versus 86.7%), but less smear positive patients were identified among those tested (8.0% versus 9.4%). Overall, all forms of TB cases identified per 100,000 screened were significantly higher in the >24-hour cough approach at OPD (92.7 for cough >24 hour versus 82.7 for cough >2 weeks ), and even higher in diabetics (364), among contacts (693) and PLHIV (995). NNS (95% Confidence Interval) varied from 100 (93-109) for PLHIV, 144 (112-202) for contacts, 275 (197-451) for diabetics and 1144 (1101-1190) for OPD attendants. About 80% of the TB cases were detected in general OPDs. Despite the intervention, notifications trends were similar in the intervention and control areas.

**Conclusion:**

The >24-hour cough approach yielded more TB cases though required TB testing for a larger number of patients. The yield of TB cases per 100,000 population screened was highest among PLHIV, contacts, and diabetics, but the majority of cases were detected in general OPDs. The intervention had no discernible impact on general case notification.

## Background

Ensuring early detection of tuberculosis (TB) cases is one of the key components of the End TB Strategy [1]. It is estimated by the World Health Organization (WHO) that there were 10.4 million incident cases of TB globally in 2015, and 1.8 million deaths due to TB [2]. Undetected active TB cases, as well as the pool of persons with latent TB infection which consists of a third of the human population, serve as an infectious reservoir for potential new cases, thereby posing a challenge to TB elimination [3]. Identification of TB cases usually depends on symptomatic patients voluntarily reporting to the health facility for diagnosis. Usually a history of cough for 2 or more weeks, with or without other TB suggestive symptoms, is the criterion used to identify people to be tested for TB. However, using this method may be limited by factors such as patient health seeking behaviour, health worker alertness and low sensitivity. Some individuals may not have TB suggestive symptoms at all, or may have less prominent symptoms that fail to elicit attention for testing for TB. Therefore, diagnosis of these cases is potentially missed or delayed with the risk of sub-optimal treatment outcomes, health sequelae and continued transmission of TB in health facilities and the general population [4, 5].

Diagnostic delays and low TB case notification pose important challenges, prompting the need to explore interventions that increase TB case detection. In implementing these interventions, however, it is pertinent that they are cost effective and targeted at selected risk groups. Additionally, it is necessary to take into consideration the potential yield of TB cases, benefits, and harms, as well as the feasibility and costs [4].

HIV clinics rank high among the settings for increased yield of TB cases due to the high risk of TB among people with HIV [6, 7]. Similarly, studies among diabetics have shown that the risk of developing TB is higher among persons with diabetes compared to non-diabetics [8, 9]. Contacts of TB cases are another risk group; data from multiple studies from low and middle income countries showed pooled prevalence of 3.1% active TB in all contacts [10].

Outpatient departments (OPD) of health facilities are feasible settings for TB symptoms screening [6, 11]. Patients presenting themselves at the health facility, although not constituting a specific TB risk group, constitute a “captive audience” requiring limited logistic arrangements compared to the labour-intensive case finding methods employed in non-facility based settings.

The 2007 comprehensive review of the Ghana National TB Program (NTP) highlighted low TB case detection as a challenge to TB control in Ghana [12]. With case detection estimated at 27% for all TB cases and 37% for smear-positive cases in 2008, the National Tuberculosis Health Sector Strategic Plan for Ghana (2009-2013) clearly identified TB case detection as one of the areas for intervention [13]. With support from WHO and Canadian International Development Agency (CIDA), the Ghana NTP subsequently implemented a provider-initiated enhanced TB case finding strategy in the capital Accra. The selection of Accra for the initiative was because of proximity to facilitate oversight and monitoring of activities by the national office of the NTP which is located in Accra. This was done under programmatic settings among attendants of general outpatient departments (OPD), HIV clinics, diabetes clinics and contacts of identified TB cases to augment TB case detection [14]. Two approaches which used different durations of cough and other TB suggestive symptoms were used to identify patients for sputum smear testing for TB.

While multiple studies have been published on screening for TB cases in different settings and countries, there is very little in the literature on enhanced TB case detection efforts in Ghana [15, 16]. The first objective of this paper was to compare the yield of the two different approaches used in two sets of general OPD clinics in Accra, with one of the screening approaches using a shorter duration of cough as well as other TB suggestive symptoms. The second objective was to compare the yield from the four groups; namely general outpatients, PLHIV, diabetics and contacts, using the approach with the shorter duration of cough and other TB suggestive symptoms. Finally, as a third objective, case notification trends in Accra were compared to those of a control area.

## Methods

This study is a retrospective analysis comparing the yield of TB cases using two different approaches to identify people eligible for TB testing from July of 2010 to December 2013. The approaches were implemented as part of an enhanced TB case finding intervention in Accra Metropolis, the largest city and capital of Ghana, located in the Greater Accra Region (GAR). The following criteria were used to select public health facilities to participate in the intervention: availability of TB microscopy services and functioning DOTS centres, large OPD clientele and capacity to implement the intervention under programmatic settings which translated into the facility management indicating ability to implement the intervention activities in the existing setting using the existing staff. Eleven major public health facilities in Accra, some having and others not having separate independently-ran HIV and diabetic clinics in addition to the general OPD services, fulfilled the criteria and were selected to participate in the intervention. OPD attendance ranged from 100 per day in the smallest facility to 500 per day in the largest facility. The intervention was implemented in the outpatient departments in ten facilities as well as HIV clinics and diabetic clinics that were operating in these facilities, but in the eleventh facility, the intervention was implemented in only the HIV clinic. These 11 facilities made up 24% of the 46 TB diagnostic facilities in Accra, but accounted for approximately 70% of cases reported in Accra city and 53% of TB cases reported in Greater Accra Region in 2009. Accra residents as well as the residents of the neighbouring districts in GAR, who flock into the city during the day to transact various activities including work and educational pursuits, patronize these facilities. To facilitate ownership and buy-in, management and all health care staff of the facilities were sensitized about the modalities of intervention. Standard operating procedures (SOPs) were developed to guide operations at the facilities and health care staff in the OPD, consulting rooms, laboratory and TB DOTS centres who were directly involved in the implementation of the initiative were trained in their use. Tools that were produced to track data included registers for contact tracing, presumptive TB patients, PLHIV screened for TB and presumptive TB referral forms and screening tool for the two screening approaches.

### Screening methods

In the first approach, assigned to 7 general OPDs, the history of cough of two or more weeks with or without other TB suggestive symptoms was elicited from all patients, regardless of the presenting symptoms, by the attending OPD nurse responsible for taking vital signs. If the patient affirmed a cough of 2 weeks or more, this was indicated on the patient’s folder/OPD treatment card to alert the clinician. The OPD nurse then filled a sputum request form for the patient, who was then sent to the laboratory for the first collection of sputum specimen. It was ensured that such patients kept their place in the queue to see the clinician. Subsequently, during the consultation, the clinician would conduct a thorough clinical examination to assess for extra-pulmonary TB in addition to making a diagnosis for the patient’s presenting symptoms. The clinician would then refer the patient to the laboratory for the second sputum smear examination, even when extra-pulmonary TB was presumed.

**Fig. 1:**
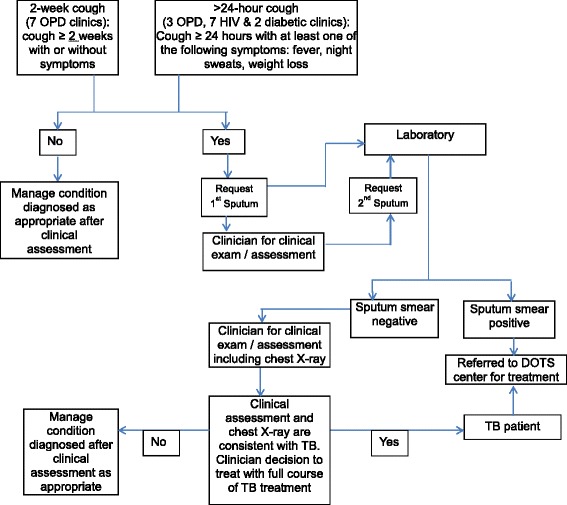
Algorithm for diagnosing TB among outpatient attendees in 11 health facilities in Accra

The second approach was assigned to 3 general OPDs, 7 HIV clinics - one of which was in a tertiary hospital - and 2 diabetes clinics, using a similar process. The difference in the second approach was that the patients were asked for a history of cough of >24 hours, as well as a history of any of the following TB suggestive symptoms: fever, weight loss, and drenching night sweats. See Fig. 1 for the diagnostic algorithm. The assignment of approaches among the clinics was purposely done in such a way that all the HIV and diabetes clinics used the second approach, hereafter referred to as >24-hour cough approach. This was in consideration of improving identification of TB in those patients who may not have the typical prolonged cough associated with TB [17]. The main reason for the selection of the three facilities to implement the >24 hour-cough approach in their OPD was because they had a relatively larger OPD clientele and it was expected that their laboratories would be able to handle the potentially larger volume of samples for sputum examination expected given the criteria of >24 hours of cough and other TB suggestive symptoms. While the patient population in the two sets of facilities was likely similar, it is important to note that the intervention was not implemented as a trial. Therefore, consideration was not given to baseline characteristics of the patient population at the facilities that could pose as potential confounding factors. We compared the yield from the two different approaches used in two sets of general OPD clinics in Accra and also compared the yield between the four groups, namely general outpatients, PLHIV, diabetics and contacts using the >24-hour cough approach.

Contact investigation for TB was not a routine practice of the facility staff. It was therefore implemented as one of the case finding interventions with the pool of people screened being contacts of TB cases, in contrast to patients attending the respective clinics for the other groups. Index TB patients from all facilities, with the exception of one that cited inadequate logistics to carry out contact investigations, were invited to list their contacts. Depending on the preference of the index patient, contacts identified were either screened during home verification of the index patient before treatment initiation, or at the health facility while accompanying the index patient. The screening approach in use at the facility of the index patient was employed for the screening of contacts. Contacts presumed to be TB cases followed the standard diagnostic algorithm.

At the time of the intervention, Ziehl Nielsen staining method was used in the diagnosis of TB. A diagnosis of sputum smear positive TB (SS+ve) was made when at least one acid-fast bacilli (AFB) was detected in 100 fields in one out of two slides. A diagnosis of smear negative pulmonary TB was made only after the smear negative sputum result had been followed up with clinician assessment and chest x-ray with findings consistent with TB coupled with the clinician decision to treat with a full course of TB treatment.

### Data collection and analyses

The main source of data for these analyses was the National Tuberculosis Control Program (NTP) database. The following data from the participating facilities was submitted to the NTP by the institutional TB coordinator on a quarterly basis over the period of the intervention: number of people verbally screened at the general OPD, diabetes clinics, HIV clinics and among contacts of index TB patients, number of presumptive TB patients identified among those verbally screened and number of people tested/evaluated for TB disease among those identified as presumptive TB patients and number of people diagnosed with all forms of TB and SS+ve TB. The data was cross-checked during periodic monitoring and supervisory support visits to the facilities by the Accra Metropolis Health Directorate TB team and NTP staff. The quarterly figures from the two different screening approaches used in the OPDs were plotted to show the trend of those screened, identified with TB suggestive symptoms, tested, and the yield of TB cases over the period of the intervention. The proportion of all forms of TB and SS+ve cases among the numbers screened, the presumptive TB cases and those tested for TB and the number needed to screen (NNS) to identify one SS+ve case, as well as all forms of TB for the two different approaches used in the two sets of general OPD clinics, HIV clinics, diabetes clinics and contact investigations were calculated. Two-sample tests of proportion were used to determine the 95% confidence intervals for these proportions to enable comparison between the two approaches used in the two sets of general OPD clinics in Accra and across the four groups, namely general outpatients, PLHIV, diabetics and contacts, to identify significant differences. STATA Data Analysis and Statistical Software version 12 was used for the analysis. For the third objective of the paper, the comparison of TB case notification trends at the population level, Greater Accra Region (GAR), in which Accra is located, was assessed as the evaluation population [18]. Like many major cities, the city of Accra is a congregating hub for residents in surrounding districts who come into the city daily for a myriad of activities including accessing health care in the city’s facilities. A series of re-demarcation of the districts in GAR has resulted in some residents originally in the geographic region of Accra being assigned to new or other districts in Greater Accra Region, and in some instances residents from the districts bordering Accra have been reassigned to the Accra population [19]. The potential of a spill over effect from the fluid population and the changes in population figures from the re-demarcation exercise necessitated the use of a larger evaluation population and geographic area in order to avoid distortions in the measurements of intervention effects [18]. Ashanti Region, with similar characteristics to GAR, was selected as the control population to compare with GAR. Kumasi, the second largest city in Ghana, is located in Ashanti Region and shares a similar profile with Accra city in population, human resource for health capacity, health infrastructure and economic activities. It also has residents commuting daily from neighbouring districts to the city for various endeavours, creating the potential for similar spill over effects [20]. In the same vain, a demarcation exercise in Ashanti Region resulted in changes in Kumasi’s population and geographic spread. In summary, because of the risk of distortion from the demarcation exercises in Accra and Kumasi and spillover effect from fluid populations, we decided to compare notification data from Greater Accra Region and Ashanti Region instead of comparing notification data from Accra and Kumasi. Quarterly notification rates (for all forms of TB and smear positive TB) per 100,000 population were plotted using Microsoft Excel 2010, using figures obtained from the NTP and Ghana Statistical Service for the period 2008 to 2013 for the 2 regions. A linear-trend line was drawn through the quarterly historical TB notification data during the baseline (the first quarter of 2008 up to the second quarter of 2010) to project TB notification expected during the intervention period for Greater Accra Region and for Ashanti Region. A linear-trend line was also drawn through the actual TB notification data during the intervention period (third quarter 2010 to fourth quarter 2013) for each region. The graphs showed how the two linear-trend lines compared to each other in the intervention area Greater Accra and in the control area Ashanti Region.

### Ethical consideration

Ethical clearance for the study was obtained from the Ghana Ministry of Health Research Division Ethical Review Board. Permission was also sought from the NTP and the participating facilities to use the data for the study. The data used in the analyses did not involve personal identifiers, but confidentiality was nevertheless maintained.

## Results

During the implementation period, out of the reported 2,954,057 persons screened in the various clinics in participating facilities, approximately 1 out of 100 (24,562) were identified as having symptoms suggestive of TB (Table 1). About 90% (21,890) of these presumptive TB cases were tested for TB. Among these 21,890 presumed TB cases tested, 84.3% were from OPD, 11.9% from the HIV clinic, 2.0% from the diabetes clinic and 1.7% from contacts investigation. Overall, 3,162 TB patients (all forms) were identified, with 79.7% from the OPD, 18% from the HIV clinic, 0.8% from the diabetic clinic, and 1.5% from the contact investigation. Among the TB patients, 57.9% (1,833) were sputum smear positive.

**Table 1:** Results from TB case finding activities in clinics in Accra Metropolis from July 2010 to December 2013

	OPD			HIV	Diabetes	Contacts	Total
Indicators	2-week cough	>24-hour cough	Total	>24-hour cough	>24-hour cough	Total	Total
Number of facilities	7	3	10	7	2	10	
(A) Number of people screened	1,522,297	1,360,846	2,883,143	57,265	6,866	6,783	2,954,057
(B) Number of presumptive TB patients identified	9,648	11,211	20,859	2,751	495	457	24562
(C) Number of people tested/evaluated for TB disease	8,358	10,100	18,458	2,610	441	381	21890
(D) Number of people diagnosed with all forms of TB	1,259	1,261	2,520	570	25	47	3162
(E) Number of people diagnosed with SS+ TB	787	803	1590	205	14	24	1833
% presumptive TB cases among those screened (B/A)	0.63%	0.82%	0.72%	4.80%	7.21%	6.74%	0.83%
95%CI	(0.62-0.65)	(0.81-0.84)	(0.71-0.73)	(4.63-5.00)	(6.60-7.82)	(6.14-7.33)	
% people tested for TB among presumptive TB patients (C/B)	86.6%	90.1%	88.5%	94.9%	89.1%	83.4%	89.1%
95%CI	(86.0-87.3)	(89.5-90.6)	(88.1-88.9)	(94.1-95.7)	(86.3-91.8)	(80.0-86.8)	
% SS+ TB patients among those tested (E/C)	9.4%	8.0%	8.6%	7.9%	3.2%	6.3%	8.4%
95%CI	(8.8-10.0)	(7.4-8.5)	(8.2-9.0)	(6.8-8.9)	(1.5-4.8)	(3.9-8.7)	
patients with all forms of TB among those screened (D/A) per 100,000	82.7	92.7	87.4	995.4	364.1	692.9	107.0
95%CI	(78.1-87.3)	(87.6-97.8)	(84.0-90.8)	(914.1-1076.7)	(221.6-506.6)	(495.5-890.3)	
SS+TB patients among those screened (E/A) per 100,000	51.7	59.0	55.1	358.0	203.9	353.8	62.1
95%CI	(48.1-55.3)	(54.9-63.1)	(52.4-57.9)	(309.1-406.9)	(97.2-310.6)	(212.5-495.1)	
% SS+ve TB patients among total number of TB patients (E/D)	62.5%	63.7%	63.1%	36.0%	56.0%	51.1%	58.0%
95%CI	(59.8-65.2)	(61.0-66.3)	(61.2-65.0)	(32.0-40.0)	(36.5-75.5)	(36.8-65.4)	
Number Needed to Screen to find one TB patient all forms (NNS1) (A/D)	1209	1079	1144	100	275	144	934
95%CI	(1145-1280)	(1022-1142)	(1101-1190)	(93-109)	(197-451)	(112-202)	
Number Needed to Screen to find one SS+TB patient (NNS2) (A/E)	1934	1695	1813	279	490	283	1612
95%CI	(1808-2080)	(1584-1821)	(1727-1908)	(246-324)	(322-1029)	(202-471)	

>2-week cough – screening approach using a history of cough of 2 or more weeks with or without other TB symptoms; >24-hour cough – screening approach using a history of cough of >24 hours as well a history of any of the following symptoms fever, weight loss and drenching night sweats. 95% CI – 95% Confidence intervals

### Quarterly variations

**Fig. 2:**
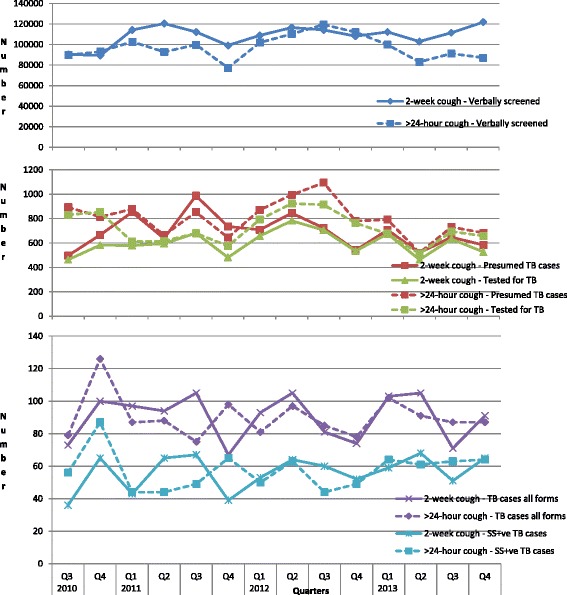
Number of people verbally screened for TB, identified as presumptive TB, tested for TB, diagnosed with all forms of TB and diagnosed with sputum positive TB identified by the quarter from third quarter 2010 to fourth quarter 2013 for 2-week cough and >24-hour cough approaches in Accra Metropolis facilities

The TB cases detected ranged from 170.8 per 100,000 screened in the fourth quarter of 2010 to 73.8 per 100,000 in the fourth quarter of 2012. Figure 2 shows the trend of the number of people verbally screened, identified as presumptive TB, tested for TB, and diagnosed with TB by quarter, from the third quarter of 2010 to the last quarter of 2013 for the two different screening approaches from the OPD clinics. While fluctuations were observed in these parameters over the period, there was no clear-cut pattern over the course of time. Linear-trends lines for the respective graphs showed that while there was an increasing trend among those verbally screened over the course of the intervention, a decreasing trend was generally identified for the number of presumptive TB cases identified and for numbers tested.

### Yield from clinics

A comparison of the 2 approaches used in the general OPD setting showed that in the >24 hour-cough approach, significantly more presumptive TB cases were identified among general outpatients (0.82% versus 0.63%, p=0.0000). Also, more patients were tested (90.1% versus 86.7%, p=0.0000) and fewer smear positive patients were identified among those tested (8.0% versus 9.4%, p<0.007) (Table 1). Overall, the rate of TB cases (all forms) identified among the outpatients screened was higher in the >24 hour-cough approach (92.7 per 100,000 versus 82.7 per 100,000, p=0.004). More patients needed to be screened to identify one TB patient in the 2-week cough approach (NNS=1209, 95% confidence interval (95%CI) 1145 - 1280) compared to the >24 hour-cough approach (NNS=1079, 95%CI 1022 - 1142). The differences between the 2 approaches in all of the above-mentioned indicators were statistically significant. However, the proportion of SS+ve TB diagnosed among all forms of TB did not differ between the two approaches.

Approximately 7% of those verbally screened in the diabetes clinic and contacts of index patients were identified as presumptive TB cases compared to about 5% in the HIV clinic. In the various groups, over 80% of people identified to be presumptive TB cases were tested. However, the HIV clinic had the highest proportion of presumptive TB cases being tested for TB (94.9%), as well as the highest proportion of those tested being diagnosed with TB (21.8%). HIV clinic attendees had the lowest proportion of cases confirmed with sputum smear microscopy (36%). Rates of TB among those screened were also highest among the HIV patients (995 per 100,000), followed by contact investigation (693 per 100,000). Consequently, the number of people needed to screen (NNS) to identify one TB case was lowest at100 for HIV patients, followed by contacts at 144.

### Evaluation versus control area

**Fig. 3:**
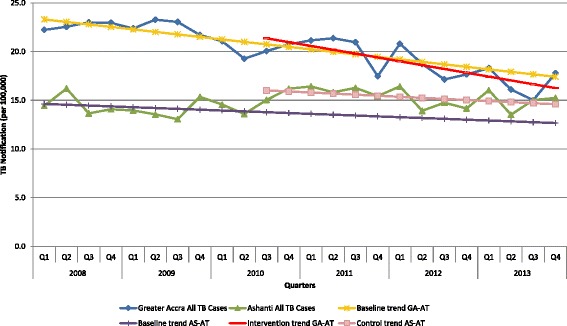
Quarterly notification rates of all TB cases for Greater Accra and Ashanti Regions with linear-trend lines from 2008 to 2013. Q: Quarter, GA-AT: Greater Accra all TB cases, AS-AT: Ashanti all TB cases. Baseline trend – refers to the linear-trend line drawn to project TB notification expected during the intervention period for both regions using quarterly historical TB notification data from Greater Accra and Ashanti Regions during the baseline period (first quarter of 2008 up to the second quarter of 2010) before the intervention started. Intervention trend – refers to the linear-trend line drawn through the actual TB notification data from Greater Accra Region during the intervention period (third quarter 2010 to fourth quarter 2013). Control trend – refers to the linear-trend line drawn through the actual TB notification data from Ashanti Region during the period (third quarter 2010 to fourth quarter 2013)

**Fig. 4:**
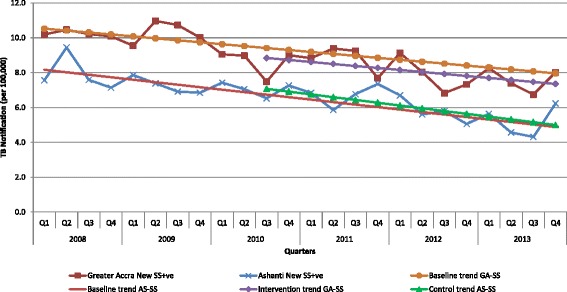
Quarterly notification rates for sputum smear positive cases for Greater Accra (GA) and Ashanti (AS) Regions with linear-trend lines from 2008 to 2013. Q: Quarter, GA-SS: Greater Accra sputum smear positive cases, AS-SS: Ashanti sputum smear positive cases. Baseline trend – refers to the linear-trend line drawn to project TB notification expected during the intervention period for both regions using quarterly historical TB notification data from Greater Accra and Ashanti Regions during the baseline period (first quarter of 2008 up to the second quarter of 2010) before the intervention started. Intervention trend – refers to the linear-trend line drawn through the actual TB notification data from Greater Accra Region during the intervention period (third quarter 2010 to fourth quarter 2013). Control trend – refers to the linear-trend line drawn through the actual TB notification data from Ashanti Region during the period (third quarter 2010 to fourth quarter 2013)

Both projected TB notification and the actual TB notification for all forms of TB and smear positive TB during the intervention period showed a downward trend in Greater Accra Region (Figs. 3 and 4). However, the actual notification data for smear positive TB cases was less than the projection using the historical data. For Ashanti Region, the control region, projected notification data for all forms of TB and smear positive TB using historical data for the projection also showed a downward trend. However, while actual notification data for smear positive TB during the intervention period was similar to the figures projected from historical data, more TB cases (all forms) were reported in Ashanti Region compared to the projected data. In other words, over the period of the intervention, more TB cases (all forms) were identified among the control population (Ashanti Region), while fewer SS+ve cases were identified in the intervention population (Greater Accra Region) compared to projected figures using historical data.

## Discussion

Various TB case finding strategies across different population groups globally have been implemented as a means of diagnosing undetected TB cases that would otherwise be difficult to identify relying only on symptomatic patients reporting to the health facility for diagnosis [21]. The yield of TB cases is affected by several factors including the screening and diagnostic methods, setting and the population being screened, which could range from those considered to be at high risk for TB to the general population. The Ghana NTP implemented a TB case finding intervention across four different groups: OPD attendants, PLHIV, diabetics, and contacts of TB cases. Among the OPD attendants, two screening approaches, which differed on duration of cough, were used. As expected, more people with presumptive TB were identified and tested for TB among the OPD clinics using the >24 hour-cough screening approach, and comparatively fewer numbers of people needed to be screened to detect one TB case (all forms). Across the four groups, the number that needed to be screened to identify a TB case was lowest among PLHIV and highest among the OPD attendants. Despite implementing this initiative, the decreasing trend in the TB notification for all forms of TB noted in the preceding two years before the start of the intervention continued. A similar phenomenon was noted in the control population.

Screening by using more sensitive methods results in an increase in the pool of presumptive TB cases from which actual cases can be identified, because the net is cast wider. This is, however, at the expense of specificity [22]. It was therefore not surprising that our study showed that compared to the OPD attendees with cough of 2 weeks or more, the OPD attendants with a shorter duration of cough yielded a higher proportion of candidates for TB testing but a lower proportion of TB cases among those tested. Yet our overall yield of 0.72% of OPD attendees identified as presumptive TB cases to be tested for TB was quite low when compared to the 2.6% to 3.5% range found in studies on the yield of potential TB cases among OPD attendees in Tanzania and Kenya [23–25]. There could be a number of reasons for this marked difference. For one, different screening criteria were used. For another, unlike strictly supervised study settings with screening conducted over shorter periods of time our screening occurred over a period of three and a half years and under programmatic settings with inherent challenges. It is therefore possible that the yield could have been higher since under the programmatic conditions, there may have been gaps in following all steps in the algorithm possibly contributing to missed opportunities to screen all patients to ensure that all presumptive cases identified underwent sputum smear microscopy.

Given the higher risk of TB among PLHIV and diabetics and by using the screening criteria of cough of any duration and at least one TB suggestive symptom, we found higher proportions of presumptive TB cases among the attendees in the HIV and DM clinics than in the OPD [7, 8]. It was noted that presumptive TB cases among PLHIV had the highest rate of testing for TB. This is indicative of good adherence to the set guidelines, requiring PLHIV with symptoms suggestive of TB to be investigated [26].

In our study the proportion with sputum smear positive results of those tested from the OPD (8.6%) fell within the range of what was found in two studies from Tanzania (6.1%) and Ethiopia (13.5%) [27, 28]. The variation could be due to the differences in settings (a tertiary facility in Tanzania and 5 public and private health facilities in Ethiopia) and the different durations of data collection. The rate of sputum smear positive results among PLHIV patients tested for TB was similar to what was found in the study by Seni and colleagues in Tanzania [27].

The proportion of sputum smear positive TB cases among all forms of TB varies in different reports and may be related to the population studied, the setting, the sensitivity of the diagnostic method and microscopy quality assurance issues [29–35]. Consequently the diverse circumstances and populations studied contribute to the range of 31.6% to 77% found in studies from different parts of the world [29–35]. The proportion of SS+ve TB among the various categories of patients in our study fell within this range. The finding of the PLHIV TB patients having relatively lower prevalence of SS+ve positive is not out of place, since this falls in line with studies reporting higher prevalence of sputum smear negative TB in PLHIV [36–38].

The ranking of the number of TB cases identified per 100,000 populations of the various groups in our study resonates with what others have found, with OPD attendees having the lowest and PLHIV the highest and more than 10 times the figure for the OPD attendees [9, 39]. Overall, slightly more TB cases (all forms) were identified among the outpatients screened in the >24 hour-cough approach (92.7 per 100,000 outpatients versus 82.7/100,000). Although the difference was significant, it is important to consider the implications of the increased burden on the laboratory having to test so many presumptive TB patients. The numbers needed to screen to find one TB patient (NNS) for all the categories of patients was in sync with what one would find in a low to moderate TB incidence country like Ghana [15].

The discovery that the case finding intervention did not demonstrate an increase in TB case notification in the intervention population compared to the comparator and even showed a downward trend compared to historical data was unexpected. It is possible that the number of extra cases was too small to see an effect. Another possibility is that some of the TB patients detected during the program would otherwise also have been detected though perhaps a bit later. It is also important to note that the intervention was facility-based and used symptoms screening to identify potential TB cases for testing, which also limited its ability to identify TB patients not exhibiting these TB suggestive symptoms and those not accessing the facilities for care. Prevalence surveys have demonstrated that 50% or more of those with bacteriologically confirmed TB may not have symptoms commonly used to presume TB [4, 40]. Considering that the 2013 TB prevalence survey in Ghana showed an estimated TB prevalence of 290 cases per 100,000 which was more than quadruple the WHO estimate at the time of the project, a lot more needs to be done to improve case finding among the general population and groups at high risk of contracting TB including miners, PLHIV and diabetics, prisoners and contacts of TB patients. [41, 42] Some of these key groups may also not access health care regularly and therefore may not be reached in interventions such as these which are facility based. As reiterated by the End TB Strategy, it is imperative to ensure universal access to early and accurate diagnosis of TB which among others includes education to trigger care seeking among those with symptoms suggestive of TB and screening among high risk groups. [42] In employing these active and enhanced case finding methods, there is a need to scale up the use of more sensitive diagnostic methods beyond sputum smear microscopy that include new molecular diagnostics and employ additional screening tools beyond symptom screening such as chest X-ray to identify other forms of TB including extra-pulmonary and bacteriologically negative forms as well as TB in children [21, 42].

Since the projection from the baseline historical data indicated a downward trend similar to the decreasing TB case notification nationally over the intervention years, it is also possible that there might be some underlying programmatic constraints contributing to fewer cases being detected [41]. This could be a subject for further investigation. Contrary to study settings in which there is meticulous supervision, monitoring and measures put in place to ensure adherence to protocols, this intervention was implemented under programmatic conditions.

There are some limitations to this study. Since it was a retrospective assessment of an intervention that was not implemented under rigorous trial conditions, some elements of bias, such as the assignment of the screening approach to the facilities, could have been introduced in the execution of the intervention. Secondly, validation of the diagnosis of TB cases was not possible. Finally, the study did not explore the possible events and prevailing circumstances that may have affected the outcomes and thrown light on some of the findings, such as suspension of OPD services that occurred as a result of industrial actions by health workers or shortage of diagnostic reagents during the intervention period. Despite these drawbacks, to our knowledge this study is the first in Ghana to assess the yield of TB cases from symptomatic screening of different categories of patients. Further study building on the finding of this paper should explore treatment enrolment and outcomes of the TB cases identified from the different settings.

## Conclusion

In this study the screening approach using a shorter duration of cough (>24 hours) had a somewhat better yield of TB cases and appears feasible for implementation. The increased workload on the laboratory, however, warrants further study to assess whether this is outweighed by the higher number of TB cases identified. This study reiterated that the yield of TB cases was highest among PLHIV, contacts of TB patients and diabetics screened but the vast majority of cases were detected in general OPDs. Though in Ghana screening of PLHIV for TB is being implemented to an appreciable extent in HIV clinics, it is important to ensure that it is being done systematically. Screening of contacts of TB cases and diabetics has been virtually non-existent. Since it is already a policy in Ghana to undertake home verification of TB cases before the initiation of treatment, tagging along screening of the contacts of the TB cases during these home visits could facilitate the identification of potential cases in this high risk group. Greater collaboration between the NTP and the Non-Communicable Disease Control Program could facilitate the introduction of TB screening in all diabetes clinics. When considering a TB screening program, it is essential to simultaneously look at the overall health system functions and enhance capacity to facilitate early detection. This would involve ensuring that more sensitive screening and diagnostic tools such as chest X-rays (CXR) and Gene Xpert (GXP) are available where needed throughout the system. The NTP is rolling out programs to further improve case detection among risk groups and including the deployment of GXPs and the launch of a digital X-ray project in health facilities [41]. Considering that the study could not demonstrate any impact on overall case notification, further research is needed to assess the impact of the introduction of these initiatives which use more sensitive methods for screening and diagnosis of TB on yield and notification.

While the study could not demonstrate any impact on overall case notification, in view of the significant pool of TB cases yet to be diagnosed sole reliance on identifying TB among patients presenting with TB suggestive symptoms or those accessing care at health facilities may limit timely diagnosis creating the conditions for disease transmission and worse outcomes.

## Abbreviations

AS: Ashanti
CIDA: Canadian International Development Agency
CXR: Chest X-ray
DM: Diabetes mellitus
ECF: Enhanced case finding
GA: Greater Accra
GXP: Gene Xpert
HIV: Human Immuno-deficiency Virus
NNS: Number needed to screen
NTP: National Tuberculosis Control Program
OPD: Out-patient department
PLHIV: Persons living with HIV
SS+ve: Sputum smear positive
TB: Tuberculosis
WHO: World Health Organization
